# Histopathologische Diagnostik intestinaler Motilitätsstörungen: Morbus Hirschsprung und andere Differentialdiagnosen

**DOI:** 10.1007/s00292-025-01514-x

**Published:** 2025-12-03

**Authors:** Thomas Menter

**Affiliations:** 1 Pathologie, Institut für Medizinische Genetik und Pathologie, Universitätsspital Basel, Basel, Schweiz; 2 Konsiliardienst intestinale Motilitätsstörungen, Institut für Medizinische Genetik und Pathologie, Universitätsspital Basel, Schönbeinstraße 40, 4031 Basel, Schweiz

**Keywords:** Aganglionose, Chronische intestinale Pseudoobstruktion, Ganglionitis, Leiomyositis, Enzymhistochemie, Aganglionosis, Chronic intestinal pseudo-obstruction, Ganglionitis, Leiomyositis, Enzyme histochemistry

## Abstract

Der Morbus Hirschsprung/Aganglionose des Kolons ist die bekannteste Form intestinaler Motilitätsstörungen insbesondere bei Kindern. In diesem Übersichtsbeitrag, der im Rahmen der Fachsitzung der AG Kinderpathologie gehalten wurde, gibt der Autor einen Einblick in die histopathologische Diagnostik des Morbus Hirschsprung inklusive der technischen Herangehensweise an die Biopsien. Daneben stellt der Autor verschiedene Differentialdiagnosen der chronischen intestinalen Pseudoobstruktion (CIPO) vor, zu deren Erkennung die histopathologische Diagnostik beitragen kann. Dies umfasst u. a. die Hypoganglionose, die intestinale neuronale Dysplasie, die Ganglionitis und die Leiomyositis.

## Abstract (en)

Hirschsprung disease/aganglionosis of the colon is the most common form of intestinal motility disorder, especially in children. In this review article, which was presented at the specialist meeting of the German Paediatric Pathology Working Group, insight into the histopathological diagnosis of Hirschsprung’s disease, including the technical approach to biopsies, will be provided. In addition, differential diagnoses of chronic intestinal pseudo-obstruction (CIPO), which can be identified with the help of histopathological diagnostics, are presented. These include—among others—hypoganglionosis, intestinal neuronal dysplasia, ganglionitis, and leiomyositis.

## Hinführung zum Thema/Einleitung

Der Kinderchirurg Harald Hirschsprung, entstammend einer nach Kopenhagen emigrierten deutschstämmigen jüdischen Familie, beschrieb systematisch 1886 das Megacolon congenitum – den Morbus Hirschsprung – und legte so die Basis für die Klassifikation kindlicher Motilitätsstörungen, ein Jahr später beschrieb er auch die Pylorusstenose bei Säuglingen.

In den letzten 140 Jahren konnte das Spektrum intestinaler Motilitätsstörungen sowohl in Bezug auf die Klinik als auch auf die Ätiologie deutlich ausgeweitet werden. Bei vielen Differentialdiagnosen leistet die histopathologische Aufarbeitung wertvolle Dienste, dies schlug sich nicht zuletzt in der 2009 veröffentlichten London-Klassifikation für gastrointestinale neuromuskuläre Pathologien nieder [13].

In diesem Übersichtsbeitrag möchte ich neben einem vertieften Einblick in die histopathologische Hirschsprung-Diagnostik das Augenmerk auch auf andere Differentialdiagnosen von Motilitätsstörungen bei Kindern und Erwachsenen richten, bei denen die Pathologie zur Diagnosefindung beitragen kann, um so eine optimale Diagnostik und eventuell auch Therapieansätze der oft seit Jahren unter ihren Beschwerden leidenden Patient:innen zu ermöglichen.

## Morbus Hirschsprung – Pathophysiologie und klinische Diagnostik

Der Morbus Hirschsprung, das Synonym ist die Aganglionose, ist definiert durch das Fehlen von Nervenzellen des Plexus submucosus und des Plexus myentericus in verschieden langen Abschnitten des Darms. Bereits in der 12. Embryonalwoche sollte die Wanderungsbewegung der Neuronen von der Neuralleiste durch den Darm – von Ösophagus bis zum Rektum – abgeschlossen sein. Falls dem nicht so ist, liegt das Krankheitsbild einer Aganglionose vor. Das Spektrum reicht vom ultrakurzen Morbus Hirschsprung (Länge des aganglionären Segments maximal 3 cm, teils ist auch nur die anale Sphinktermuskulatur betroffen) bis zu einer Aganglionose, die sich bis in den Dünndarm erstreckt (Synonym Jirásek-Zuelzer-Wilson-Syndrom).

Pathophysiologisch kommt es zu einer fehlenden Modulation des Parasympathikus, was zu einer gesteigerten Aktivierung der Ringmuskulatur des aganglionären Abschnitts führt [23]. Dies bedingt die typische Morphologie in der Röntgenkontrastaufnahme mit dem stenotischen aganglionären Segment und der proximalwärts nachweisbaren deutlichen Dilatation des Darms. „Klassische“ Hirschsprung-Patient:innen präsentieren sich bereits kurz nach der Geburt mit fehlendem Mekoniumabgang oder Mekoniumileus. Bei kurzstreckig ausgeprägten Aganglionosen kann die Präsentation auch erst im weiteren Verlauf erfolgen, der älteste Patient mit einer Erstdiagnose eines Morbus Hirschsprung in meiner Konsiliarpraxis war 16 Jahre alt.

## Morbus Hirschsprung – histopathologische Diagnostik

Goldstandard für die Hirschsprung-Diagnostik sind Rektumbiopsien bei 1, 2 und 4 cm, die genügend Tela submucosa enthalten sollten. Klassische Schleimhautbiopsien sind oft nicht aussagekräftig, da hier per se keine Nervenzellen des in der Tela submucosa liegenden Plexus submucosus miterfasst sind.

**Abb. 1:**
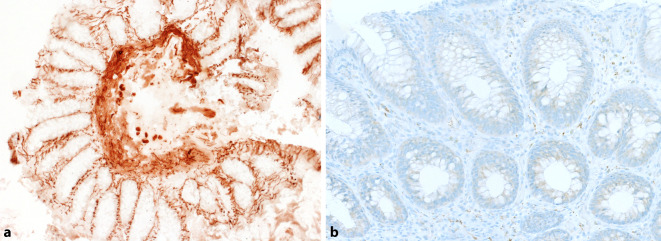
Wichtigste Befunde der Hirschsprung-Diagnostik: **a** Typische Überaktivierung des Parasympathikus in der Acetylcholinesterasefärbung mit Nachweis einer baumartigen Verzweigung der Nervenfaseräste (Acetylcholinesteraseenzymhistochemie; Vergr. 200×). **b** Immunhistochemie für den Cholintransporter, die auch die Überaktivierung des Parasympathikus zeigt (Immunhistochemie, Vergr. 200×)

Bereits in den 1970er-Jahren wurde von Herrn Prof. William A. Meier-Ruge die Enzymhistochemie für die Acetylcholinesterase vorgestellt [16]. Durch die vermehrten cholinergen Fasern des aktivierten und entsprechend ungehemmten Parasympathicus und das so vermehrt vorhandene Acetylcholin ist auch das das Acetylcholin abbauende Enzym Acetylcholinesterase entsprechend vermehrt präsent, das färberisch dargestellt werden kann (s. Abb. 1a). Der Vorteil dieser Färbung ist, dass so die Diagnose des Morbus Hirschsprung mittels eines „Positivbelegs“ gestellt werden kann: eine Vermehrung der parasympathischen Innervation. Der „Negativbeweis“, der Nachweis eines Fehlens von Nervenzellen, kann manchmal schwierig sein. Gründe hierfür sind Biopsien ohne ausreichende Tela submucosa, ein sehr heterogen aufgebauter Plexus submucosus oder auch Schwierigkeiten bei der histopathologischen Aufarbeitung der Präparate (tangentiale Anschnitte, technische Färbeprobleme etc.). Neben der gesteigerten parasympathischen Aktivität können die Nervenzellen durch verschiedene verstärkt in den Nervenzellen vorhandene Enzyme (Laktatdehydrogenase, Succinatdehydrogenase, Nitritoxidase) dargestellt werden, was auch bei einigen Differentialdiagnosen des Morbus Hirschsprung dienlich sein kann.

Ein Nachteil der Enzymhistochemie ist die Voraussetzung, dass Frischgewebe notwendig ist, da die Enzymhistochemie per se nicht am formalinfixierten Gewebe funktioniert (Degeneration der Enzyme, die die Färbereaktion auslösen). Dies stellt insbesondere für Kliniken, bei denen diese Technik nicht vor Ort angeboten wird, ein Problem dar, da dies einen Transport gefrorener Proben und so einen entsprechend teuren logistischen Aufwand bedeutet. Für das Pathologielabor stellt die Enzymhistochemie auch einen großen Aufwand dar, da sowohl Reagenzien vorgehalten werden müssen, die ansonsten nicht in der Pathologie eingesetzt werden, als auch entsprechend geschultes Personal. Zudem kommt es in den letzten Jahren durch labile weltweite Lieferketten immer wieder zu Lieferengpässen bestimmter Reagenzien, die oft nur schwer zu substituieren sind.

Die Aufarbeitung der Kryobiopsien erfolgt routinegemäß in Serienschnitten mit ca. 20 Stufenschnitten pro Objektträger. Wir fertigen hierzu serielle Schnitte à 6–8 Schnitte an, die dann nacheinander auf die verschiedenen Objektträger aufgezogen werden. Nach einer solchen Serie beginnen wir wieder beim ersten Objektträger, sodass auf jedem Objektträger Serienschnitte in verschiedenen Tiefen des Gewebes vorliegen. Für die von uns verwendeten Färbeprotokolle verweise ich auf entsprechende Publikationen [15].

Auch für den Schnellschnitt bietet sich die LDH-Färbung für die Suche nach Ganglien an, da sie dort deutlich einfacher zu identifizieren sind als im HE-Schnitt, insbesondere mögliche „pitfalls“ wie hypertrophe Nervenbündel ohne Nervenzellen oder Gefäße mit prominenten Endothelien lassen sich so zuverlässig vermeiden. Die Färbereaktion benötigt ca. 15 min und sollte so gut in die Operationsplanung der Durchzugsoperation einzubinden sein.

In 2017 wurde in der Literatur die Verwendung eines für formalinfixierten und paraffineingebetteten Gewebeantikörpers für den Cholintransporter beschrieben, der als Surrogatmarker für die Überaktivierung des Parasympathikus und somit als „Positivbeweis“ für den Morbus Hirschsprung eingesetzt werden kann [11]. Wir haben diesen Antikörper bei uns am Institut entsprechend etabliert und nutzen ihn für die Frage der Hirschsprung-Diagnostik in Fällen, in denen kein Frischgewebe für die Enzymhistochemie zur Verfügung steht (s. Abb. 1b). Auch die Cholinrezeptorimmunhistochemie kann nur bei Rektumbiopsien für die Frage nach einem Morbus Hirschsprung einsetzt werden, da sich weiter proximal im Kolon keine gesteigerte parasympathische Aktivität nachweisen lässt.

Ein anderer immunhistochemischer Ansatz ist die Färbung für Calretinin (negativ in der aganglionären Zone) und S100 (Nachweis der vermehrten Nervenfasern in der Tela submucosa) und einen Nervenzellmarker (z. B. MAP2, PGP9.5; [2, 5]). Bei diesem Ansatz bleibt jedoch das Problem des negativen Resultats für die Hirschsprung-Diagnose bestehen, da – im Gegensatz zur Färbung mit Acetylcholinesterase und dem Cholintransporter – kein positives Färberesultat für die Diagnose Morbus Hirschsprung vorliegt, was differentialdiagnostisch an ein falsch-negatives Resultat denken lassen könnte.

Ein manchmal schwierig zu interpretierender Befund ist die sog. Transitionszone. Hierbei handelt es sich um den Darmabschnitt proximal der aganglionären Zone, in dem die Ganglien des Plexus myentericus noch irregulär verteilt sind, was sich negativ auf die Peristaltik dieses Darmabschnitts auswirken kann. Die Transitionszone kann sehr variabel sein, in der Literatur werden Längen zwischen 3 und > 20 cm angegeben [9]. Die Beurteilung der Transitionszone ist eine wichtige Aufgabe im Rahmen der Schnellschnittuntersuchung, um die Länge des im Rahmen der Durchzugsoperation Kolonsegments zu bestimmen. Anstelle der oft verwendeten klassischen HE-Färbung wenden wir die enzymhistochemische Färbung mit der Laktathydrogenase an, mit der die Ganglien des Plexus myentericus deutlich einfacher zu identifizieren sind. Diese Färbereaktion dauert ca. 15 min und kann entsprechend bei der Operationsplanung berücksichtigt werden. Zudem hat sich die Aufarbeitung der Hirschsprung-Resektate in der „Swiss-roll-Technik“ bewährt, bei der die Präparate im unfixierten Zustand aufgerollt und entsprechend sowohl als Kryoblock als auch als formalinfixiertes Präparat untersucht werden können. Entsprechende Anleitungen hierzu findet sich auf der Internetseite www.hirschsprung.ch.

## Ultrakurzer Morbus Hirschsprung/Sphinkterachalasie

Eine Sonderform des Morbus Hirschsprung ist eine aganglionäre Zone mit einer Länge von < 3 cm bzw. einer nur den analen Sphinkterapparat betreffende Aganglionose. In diesem Fall lassen sich in Biopsien aus diesem Bereich keine Ganglien darstellen, jedoch finden sich in den distalsten Zentimetern des Rektums insbesondere im Plexus submucosus nur sehr wenige bis keine Ganglien, was die Diagnose erschwert. Hilfreich in diesen Fällen ist das Färbemuster der Acetylcholinesterase, das vermehrte cholinerge Fasern in der Lamina muscularis mucosae und nicht in der Lamina propria der Tunica mucosa zeigt [18]. Daneben finden sich auch hypertrophe Nervenfaserbündel in der Tela submucosa.

## Das Krankheitsbild der chronischen intestinalen Pseudoobstruktion

Die chronische intestinale Pseudoobstruktion (CIPO) ist definiert als gastrointestinale Motilitätsstörung mit rezidivierender und sich teils deutlich aggravierender Symptomatik ohne anatomisch fassbares Korrelat einer Obstruktion (Tumor, Invagination, Bride etc.; [6]). Bei Kindern wird teils auch das Akronym PIPO (pädiatrische intestinale Pseudoobstruktion) verwendet.

Die Ursachen der CIPO sind vielfältig und reichen von klar genetisch definierten Erkrankungen zu primär morphologisch definierten bis hin zu „sekundären“ Formen, die z. B. mit einer Grunderkrankung oder einer Medikamenteneinnahme assoziiert sind. Für eine Übersicht zur komplexen Ätiologie der CIPO/PIPO verweise ich auf entsprechende Literatur [3, 7, 26].

Wie eingangs erwähnt, kann sich ein Morbus Hirschsprung insbesondere mit einem kurzen bis sehr kurzen aganglionären Segment nicht mit den klassischen Zeichen des Mekoniumileus bereits im Neugeborenenalter manifestieren. Viele Kinder zeigen eine mehr oder weniger ausgeprägte Obstipationssymptomatik im Entwicklungsverlauf, sodass der Morbus Hirschsprung auch bei Kindern jenseits des Neugeborenenalters eine Differentialdiagnose der CIPO darstellen kann.

Im Folgenden möchte ich auf einige weitere Differentialdiagnosen der CIPO/PIPO eingehen, bei denen die histopathologische Begutachtung eine Rolle spielt.

## Voraussetzungen für die histopathologische Diagnostik bei der Frage nach Ursachen einer chronischen Obstipation jenseits des Morbus Hirschsprung

Vorausschicken möchte ich noch einige Bemerkungen zur Auswahl des eingesandten Materials: die meisten Pathologien der CIPO spielen sich in der Tunica muscularis propria ab, Schleimhautbiopsien, auch wenn sie Tela submucosa enthalten, sind somit für diese Fragestellungen ungeeignet. Wenn möglich, sollten eine oder mehrere Kolonvollwandbiopsien mit allen 4 Schichten der Kolonwand gewonnen werden und falls sich klinisch auch eine Beteiligung des Dünndarms zeigt, sollte auch dieser biopsiert werden [26, 28]. Die zentralisierte Aufarbeitung der Proben in entsprechend befähigten Zentren wird empfohlen [26]. Aufgrund der anatomisch bedingten strukturellen Wandveränderungen des Ileozökalpols sind dieser und auch die Appendix vermiformis für die Fragestellung einer CIPO nicht oder nur eingeschränkt verwertbar. Die Vollwandbiopsie stellt natürlich einen deutlich größeren Eingriff als die klassischen Rektumsaugbiopsien bei der Frage nach einem Morbus Hirschsprung dar, es gibt hierzu jedoch aus der Sicht der morphologischen Beurteilung keine Alternative [27].

## Hypoganglionose

Eine Verminderung der Nervenzellen des Plexus myentericus kann im Rahmen verschiedener Erkrankungen auftreten [4]. Klassisch ist ihr Auftreten im Rahmen der Transitionszone des Morbus Hirschsprung, auch kongenitale Hypoganglionose genannt. Selten tritt diese auch ohne eigentliche aganglionäre Zone auf. Erfolgt eine Resektion noch in dieser Zone, die eine Länge zwischen 3 und 20 cm haben kann, kann es nach der Operation zu einer insuffizienten Peristaltik und einer Persistenz der Obstipationssympomatik kommen, die Suche nach dem Ende der Transitionszone ist auch die Rationale für die Schnellschnittdiagnostik im Rahmen der Hirschsprungresektion. Gemäß morphometrischen Studien an Gefrierschnitten ist sowohl die Anzahl an Nervenzellen pro Ganglion (6 ± 1 vs. 15 ± 4) als auch der Abstand zwischen den Ganglien (420 vs. 170 μm) signifikant unterschiedlich [20].

**Abb. 2:**
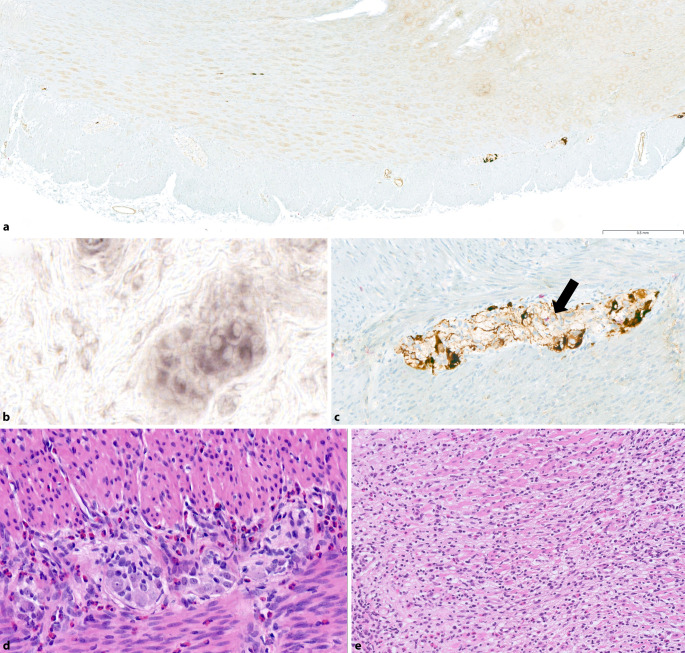
Differentialdiagnosen der chronischen intestinalen Pseudoobstruktion (CIPO): **a** Hypoganglionose: irregulär verteilte und nervenzellarme Ganglien des Plexus myentericus (Immunhistochemie für MAP2 und CD8, Vergr. 40×). **b** Intestinale neuronale Dysplasie Typ B mit Nachweis von Riesenganglien (Enzymhistochemie für LDH, Vergr. 400×). **c** Lymphozytäre Ganglionitis: in der immunhistochemischen Doppelfärbung für MAP2 (Nervenzellen) und CD8 (zytotoxische T‑Zellen) lässt sich die lymphozytäre Ganglionitis belegen (*Pfeil;* Immunhistochemie, Vergr. 200×). **d** Eosinophile Ganglionitis: Diese Diagnose lässt sich bereits HE-morphologisch sichern (HE [Hämatoxilin-Eosin], Vergr. 200×). **e** Leiomyositis mit Nachweis eines dichten lymphozytären Infiltrats in der Darmmuskulatur, es zeigt sich bereits ein beginnender Verlust glatter Muskelzellen (HE, Vergr. 100×)

Bei Erwachsenen findet sich meist die Form der „atrophischen Hypoganglionose“, auch diese ist entsprechend definiert durch eine Reduktion der Nervenzellen bezogen auf 100 mm Kolonlänge (513 ± 291 vs. 1267 ± 274) und die Anzahl der Nervenzellen pro Ganglion (2 ± 1 vs. 4 ± 1; [20]; s. Abb. 2a). Typischerweise zeigt sich auch eine sehr irreguläre Verteilung der Ganglien des Plexus myentericus, was den Verdacht auf eine Hypoganglionose erlaubt. Die atrophische Hypoganglionose ist an sich eher als Sekundärphänomen der chronischen Obstipation anzusehen, Ursache kann z. B. eine lymphozytäre Ganglionitis oder auch ischämische Veränderungen aufgrund anderer Ursachen einer CIPO sein.

## Intestinale neuronale Dysplasie

Seit ihrer Erstbeschreibung durch Prof. Meier-Ruge 1971 ist die intestinale neuronale Dysplasie ein kontroverses Thema mit Befürwortern und klaren Gegnern, die diese Entität an sich in Frage stellen [12]. Nach dem Erstbeschreiber gibt es zwei an sich vollständig pathogenetisch unterschiedliche Arten der intestinalen neuronalen Dysplasie.

Beim sehr seltenen Typ A handelt es sich um ein Defizit der sympathischen Innervierung des Darms. Die betroffenen Kinder präsentieren sich bereits kurz nach der Geburt mit abdominellen Beschwerden, Diarrhö und Meläna. Histologisch zeigt sich das Bild einer nekrotisierenden Enterokolitis mit gesteigerten parasympathischen Nervenfasern.

Der Typ B wird definiert durch „Riesenganglien“ (Ganglien mit > 7 Nervenzellen) des Plexus submucosus, diese müssen > 20 % aller Ganglien der Rektumschleimhautbiopsie ausmachen ([19]; Abb. 2b). Diese Definition beruht auf 20 µm dicken LDH-Gefrierschnitten und dem Untersuchen von mindestens 30 Serienschnitten [19]. Die intestinale neuronale Dysplasie Typ B kann im Bereich der Transitionszone eines Morbus Hirschsprung oder auch isoliert ohne begleitende aganglionäre Zone vorkommen. Letztendlich handelt es sich hierbei um eine Unreife des enterischen Nervensystems, in den meisten Fällen sollte es zu einer Ausreifung des Nervensystems mit Reduktion der Symptomatik kommen, Riesenganglien sollten jedoch immer auch als Indikatorläsion einer weiter distal gelegenen Aganglionose im Befundbericht diskutiert werden.

Auch zum Gebiet der neuronalen Dysplasie im weiteren Sinn zählt die Ganglioneuromatose. Hier sieht man zahlreiche Riesenganglien mit bis zu 30 Nervenzellen und eine deutliche Hyperplasie des submukosalen Nervenplexus. Die Ganglioneuromatose kann eine Indikatorläsion für verschiedene genetische Erkrankungen sein (multiple neuroendokrine Neoplasie 2B [MEN2B], Neurofibromatose 1, Cowden-Syndrom; [14]).

## Lymphozytäre und eosinophile Ganglionitis

Die lymphozytäre Ganglionitis ist gemäß der London-Klassifikation schon durch den Nachweis eines Lymphozyten in einem Ganglion des Plexus myentericus definiert ([13]; Abb. 2c). Zur besseren Beurteilung benutze ich hier eine immunhistochemische Doppelfärbung (MAP2-CD8), um dies nachweisen zu können, da teils der lichtmikroskopische Eindruck unklar sein kann (Fehlinterpretation glialer Zellen in den Ganglien). Die Ätiopathogenese der lymphozytären Ganglionitis ist noch nicht ganz geklärt, in erster Linie handelt es sich hierbei um eine Autoimmunerkrankung mit langsamer Progredienz. In größeren Fallserien an erwachsenen CIPO-Patienten konnte in einem Teil der Fälle eine entsprechende Ganglionitis nachgewiesen werden [24]. Bei diesen Patienten konnte die ebenfalls nachgewiesene Hypoganglionose so pathogenetisch erklärt werden.

Neben der lymphozytären Ganglionitis existieren auch Fallberichte über eine eosinophilen-dominierte Ganglionitis ([25]; s. Abb. 2d), selten ist auch ein kombiniertes Auftreten dieser Befunde beschrieben [22].

## Leiomyositis

Ein sehr selten anzutreffendes Krankheitsbild ist die Leiomyositis mit Befall der Tunica muscularis propria des Darms [8]. Die Patient:innen stellen sich oft mit akutem Abdomen bzw. Ileussymptomatik vor, teils kommt es auch zur Perforation des Darms. Histologisch zeigt sich ein dichtes T‑Zell-dominantes Entzündungsinfiltrat mit Punctum maximum in der glatten Muskulatur der Tunica muscularis propria, teils sieht man bereits einen Untergang der glatten Muskelzellen (s. Abb. 2e). Eine in dieser Konstellation auftretende Ganglionitis ist in erster Linie als sekundär anzusehen (Übergreifen des Entzündungsinfiltrats von der glatten Muskulatur auf die Plexusloge). Ätiopathogenetisch kommt in vielen Fällen ein Virusinfekt als Auslöser der Entzündung durch entsprechende Kreuzreaktionen in Frage, klassisch hierfür sind Adenovirusinfektionen [1].

Teils kann das Entzündungsinfiltrat auch durch eosinophile Granulozyten dominiert werden, in diesem Fall wird der Terminus der eosinophilen Leiomyositis verwendet.

## Myopathien

Auch viszerale Myopathien können eine CIPO-Symptomatik hervorrufen. Oft liegt bei den Patient:innen nicht nur eine Darm- sondern auch eine Blasensymptomatik vor. Morphologisch zeigen sich Irregularitäten im Wandaufbau der glatten Muskulatur der Tunica muscularis propria (z. B. Nachweis einer dritten Muskelschicht), in der Literatur sind auch degenerative Atypien oder inrazytoplasmatische Einschlüsse der glatten Muskelzellen beschrieben [10]. Mittlerweile sind verschiedene Genmutationen bei diesen Patienten beschrieben (z. B. *FLNA, ACTG2*), sodass bei Patienten mit dem histologischen Nachweis der beschriebenen Veränderungen eine genetische Beratung empfohlen werden sollte. Auch Mitochondriopathien können die Ursache für diese Leiomyopathien sein.

## Desmose

Die Desmose ist definiert durch pathologische Veränderungen des kollagenfaserigen Stützgerüsts der Tunica muscularis propria. Dadurch kommt es zu Störungen in der Koordination der Darmperistaltik und somit zu Obstipationsbeschwerden [17].

Morphologisch am auffallendsten ist ein Verlust der bindegewebigen Plexusloge des Plexus myentericus, der gut in einer Picro-Siriusrotfärbung darstellbar ist.

Das Hauptproblem bei der Beurteilung der Frage nach einer Desmose ist es, festzustellen, ob es sich bei der Desmose um primäre oder sekundäre Veränderungen (im Sinne einer „Vernarbung“ der Darmwand handelt). Der Nachweis eines normal konfigurierten Plexus myentericus legt eine primäre Desmose nahe, falls sich eine deutliche Hypoganglionose oder auch eine Ganglionitis findet, ist eher von einer sekundären Desmose auszugehen [21].

## Fazit für die Praxis

- Die Untersuchung von Rektumstufenbiopsien ist der Goldstandard für die Hirschsprung-Diagnostik.
- Neben der Enzymhistochemie kann auch mittels der Immunhistochemie sowohl das Fehlen von Ganglien als auch der Nachweis einer Überaktivierung des Parasympathikus nachgewiesen werden.
- Neben dem Morbus Hirschsprung existieren zahlreiche weitere Krankheitsbilder mit Obstipationssymptomatik, die durch entsprechende Biopsien einer klaren Diagnose zugeordnet werden können, hierzu sind jedoch meist Vollwandbiopsien notwendig, da sich die Pathologie überwiegend in der Tunica muscularis propria findet.
